# Assessment of Primary Care Physicians’ Ability to Recognize Common Skin Conditions in Saudi Arabia: A Cross-Sectional Study

**DOI:** 10.7759/cureus.81276

**Published:** 2025-03-27

**Authors:** Ahmed Abdulaziz Alsaati, Reem Abdullah Albejais, Rahaf Faisal Aldawish, Kawthar Hassan Alali, Samia Talal Abdullah Khalil, Sajjad Ibrahim Alhajji, Nouf Talal A Mleeh, Logain Alghanemi

**Affiliations:** 1 Department of Internal Medicine, Jubail General Hospital, Jubail, SAU; 2 Department of Internal Medicine, Prince Saud Bin Jalawy Hospital, Al-Ahsa Health Cluster, Al-Ahsa, SAU; 3 Department of Dermatology, King Fahad Specialist Hospital, Al-Qassim, SAU; 4 Department of Diagnostic Radiology, King Fahad Hospital, Al-Hofuf, SAU; 5 Department of Dermatology, College of Medicine, Ibn Sina National College for Medical Studies, Jeddah, SAU; 6 Department of Dermatology, College of Medicine, King Faisal University, Al-Hofuf, SAU; 7 Department of Dermatology, Faculty of Medicine, King Abdulaziz University, Jeddah, SAU

**Keywords:** dermatology, diagnosis, primary care physicians, primary healthcare, referral, treatment

## Abstract

Background

Primary care physicians (PCPs) should have adequate skills to manage common skin conditions, such as eczema, psoriasis, and vitiligo, and reduce the rate of referrals to dermatologists.

Objective

The aim of this study was to assess PCPs’ knowledge in diagnosing and managing common dermatological conditions.

Methods

This cross-sectional survey study involved PCPs in Saudi Arabia. The questionnaire consisted of three sections: demographic information, case scenarios, and questions assessing the ability to manage skin conditions. Knowledge scores were calculated by assigning one point for each correct answer. Participants rated their perceived ability on a Likert scale, ranging from 1 (totally disagree) to 5 (totally agree).

Results

Diagnostic accuracy was generally high, with between 61.5% and 89.8% of respondents correctly identifying the presented cases. However, only about 50% had effective knowledge of tinea corporis, seborrheic keratosis, acute urticaria, and tinea capitis. Higher knowledge levels correlated with older age (p<0.001), being a consultant or specialist (p<0.001), and working in primary healthcare (p<0.001). While 63.2% felt confident in managing common skin conditions, this confidence correlated weakly with overall knowledge (rho = 0.261, p = 0.002). Additionally, over 90% of PCPs agreed to refer difficult cases.

Conclusions

The knowledge of PCPs in the management of common skin conditions showed several serious deficiencies, leading to unnecessary referrals to dermatologists. There is a need for specific dermatology training programs for PCPs. Dermatology courses in medical school curricula should focus on preparing students to manage common skin conditions.

## Introduction

In general practice, skin diseases rank as the commonest cause for consultation. Factors such as environmental exposure, occupational risks, and lifestyle behaviors may influence the prevalence and presentation of dermatological conditions among patients. In Saudi Arabia, a recent meta-analysis reported pooled prevalence rates of 24%, 24.8%, and 18.5% for dermatitis, disorders of skin appendages, and skin infections, respectively [1]. A study assessing dermatological consultations at Qassim University’s dermatology clinics in Saudi Arabia found that the most prevalent skin diseases were eczema/dermatitis (19.5%), viral infections (16.6%), pilosebaceous disorders (14.4%), pigmentary lesions (11.2%), and hair disorders (7.6%) [2].

Although most skin diseases are not life-threatening, even minor skin abnormalities can be a source of psychological distress and negatively impact patients' quality of life. Visible skin lesions can lead to loss of self-esteem, depression, and negative effects on patients’ education or work [3,4].

A considerable proportion of patients with skin diseases seek consultations from primary care physicians (PCPs) [5], expecting to receive optimal care before potentially seeking expert consultation by dermatologists if their conditions require it [6]. Estimates show that consultations for skin diseases account for nearly a quarter of all consultations undertaken by PCPs [5]. Therefore, PCPs must possess adequate competence to manage common skin diseases by providing prompt treatment and decreasing the rate of referrals to dermatologists [7,8]. Consequently, PCPs and general practitioners should receive appropriate training on the management of common skin diseases to fulfill their expected role in the early detection of cases and decrease the rate of referral to dermatology clinics [1,2].

However, previous studies have shown that the knowledge and practice of PCPs and family physicians regarding the management of skin conditions may fall below the required levels. In Western India, a cross-sectional photo-based study reported that 70.5% of participants ranked their abilities to identify and treat skin conditions as "average", while one-third of the participants expressed their need to receive training on common skin conditions [9]. Another study in Yemen found that PCPs did not show adequate knowledge of common skin diseases and therapies [10]. In Abha City, Saudi Arabia, a study reported that 69.5% of the participating PCPs showed inadequate knowledge levels about common skin diseases, and 49.5% were incompetent to manage skin disorders [11].

It is critical to assess PCPs' competence in diagnosing and managing common skin conditions to identify knowledge gaps, recognize areas of strength, and guide targeted training programs to improve patient care. Therefore, the aim of this study was to evaluate PCPs' knowledge in the diagnosis and management of common dermatologic conditions and to determine if additional dermatology courses are needed to improve their understanding and management of these conditions.

## Materials and methods

Study design, area, setting, and date

A cross-sectional, survey study was conducted across Saudi Arabia. Data collection was carried out by enrolling PCPs attending primary care centers and general hospitals in Saudi Arabia. Data collection took place between September 2023 and August 2024.

Study population and target group

The study included PCPs (either family physicians or general practitioners) of all genders and nationalities from various centers in Saudi Arabia, who agreed to answer the questionnaire and those who were on duty in primary care centers during the study. We excluded PCPs who had been affiliated with primary care centers for a period of less than three months and those who refused to participate in the questionnaire.

Sample size

The sample size was 400 participants. To compensate for incomplete responses, 20% were added, so that the final sample size became 480.

Sampling technique

Participants were selected using a convenience sampling technique, and only PCPs meeting the inclusion criteria were invited to participate.

Outcome assessment

Primary Outcome

The primary outcome was to assess the PCPs’ clinical knowledge of skin lesions and their ability to manage them correctly.

Secondary Outcome

The secondary outcome was to assess PCPs’ beliefs about their ability to manage common skin disorders and the need to refer to dermatology clinics for common, non-morbid skin lesions that do not require dermatologist consultations.

Data collection

We used an electronic, structured, self-administered questionnaire to collect data. The survey contained photo-based quizzes to assess the knowledge of PCPs in diagnosing and treating common dermatological conditions. The questionnaire required no more than 10-15 minutes to complete.

The survey questions and cases were chosen based on the authors’ clinical experience and the most common skin diseases in Saudi Arabia reported by previous prevalence studies [2] to ensure relevance to primary care practice and to cover a broad spectrum of commonly encountered conditions. The questionnaire was divided into three parts: the first section was concerned with demographic and professional characteristics (age, gender, position, healthcare center affiliation, work experience, and the number of dermatological cases seen within the clinic per week); the second section contained common skin condition case scenarios (10 case scenarios with different diagnosis and treatment questions); the third and last section focused on assessing the ability of participants to manage skin conditions by providing 7 different statements and asking them how much they would agree with them. The questionnaire was independently reviewed by two experienced dermatologists.

Participants were informed about the study objectives, methodology, risks, and benefits, and they were asked to give informed consent to participate in the study upon starting to fill in the questionnaire.

Ethical considerations

The study obtained ethical approval from the Institutional Review Board of King Faisal University (KFU-REC-2023-AUG-ETHICS1012).

Data analysis

Data from completed questionnaires were collected, collated, and entered through a Microsoft Excel spreadsheet. Only the investigators had access to the participants' data. Statistical analysis was performed using the R Statistical language (version 4.4.2; R Core Team, 2024). For the computed scores, the median, interquartile range (IQR, 25th-75th percentiles), and range were calculated. For qualitative data, the variables were summarized as frequencies (count and percentage). Pearson’s Chi-square test for independence, Fisher’s exact test, or Chi-squared test for trend in proportions were used to examine the association between two categorical variables. Each correct response to knowledge questions was assigned one point, while incorrect answers received zero points. The points for all knowledge questions were summed up as the total knowledge score. Regarding the questions assessing the participants' ability to manage skin cases, the responses were assigned points from 5 (totally agree) to 1 (totally disagree). The points for all questions were summed up as the total ability score. Spearman’s rank-order correlation was run to assess the direction and strength of the correlation between the total knowledge score and confidence in diagnosing skin conditions. No multiple comparison corrections were applied, as the study focused on predefined primary and secondary outcomes rather than exploratory subgroup analyses. A p-value of <0.05 will be considered significant.

## Results

The total number of respondents to the questionnaire was 361, out of which 78 participants were excluded as their work experience in primary healthcare was less than three months. The remaining 283 respondents were included in the study. Internal consistency was assessed using Cronbach’s alpha, which had a value of 0.752 (bootstrap 95% CI: 0.717-0.781).

Approximately half of the participants belonged to the age group "30 - 50 years," 42.8% were younger than 30 years, and only 5.65% were older than 50 years. The percentage of female physicians was slightly higher than that of their male counterparts (57.6% vs. 42.4%, respectively). The participants occupied different positions in the specialty of family medicine, including consultants (15.9%), specialists (24.7%), and residents (28.27%). In addition, 31.1% of the participants were general practitioners or service residents. About two-thirds of the participants were currently affiliated with a primary healthcare center, while one-quarter were affiliated with a general hospital. Nearly half of the participants were working in healthcare centers in the Eastern province, while 26.9% were in the Mecca province. The rate of dermatological cases seen per week was less than 20 patients in 67.5% of participants, while the rate was 20-50 patients in 29.3% and above 50 patients in only 3.18% of participants (Table 1).

**Table 1:** Participants’ characteristics.

Characteristics	N = 283
Age, n (%)	
Less than 30 years of age	121 (42.8%)
30-50 years	146 (51.6%)
More than 50 years of age	16 (5.65%)
Gender, n (%)	
Female	163 (57.6%)
Male	120 (42.4%)
Current Position, n (%)	
Family Medicine Consultant	45 (15.9%)
Family Medicine Specialist	70 (24.7%)
Family Medicine Resident (PGY4/R4)	14 (4.95%)
Family Medicine Resident (PGY3/R3)	19 (6.71%)
Family Medicine Resident (PGY2/R2)	24 (8.48%)
Family Medicine Resident (PGY1/R1)	23 (8.13%)
General Practitioner/ Service Resident	88 (31.1%)
Type of Healthcare Center that you are affiliated with, n (%)	
Primary Healthcare Center	190 (67.1%)
General Hospital	72 (25.4%)
Private Hospital	20 (7.07%)
Private Clinic	1 (0.35%)
Under which province or region is your current affiliated Healthcare Center?, n (%)	
Eastern Province	145 (51.2%)
Mecca Province	76 (26.9%)
Medina Province	22 (7.77%)
Riyadh Province	18 (6.36%)
Al-Qaseem Province	17 (6.01%)
Northern Borders Province	2 (0.71%)
Al-Baha Province	1 (0.35%)
Aseer Province	1 (0.35%)
Najran Province	1 (0.35%)
Work experience in the current primary healthcare center, n (%)	
Experience of more than 3 months	283 (100.0%)
How many dermatological cases are seen within your clinic per week?, n (%)	
Less than 20 cases per week	191 (67.5%)
Between 20 and 50 cases per week	83 (29.3%)
More than 80 cases per week	9 (3.18%)

The respondents’ answers to questions that assessed their knowledge were correct in most cases, with between 61.5% and 89.8% of respondents correctly identifying the presented dermatological conditions. However, more than half of the respondents selected wrong answers regarding the diagnosis and treatment of tinea corporis (questions 13 & 14) and seborrheic keratosis (questions 19 and 20). Additionally, 47.3% and 42.4% of the respondents did not know the correct treatment for acute urticaria and tinea capitis, respectively (Table 2).

**Table 2:** Answers to knowledge questions.

Questions	Correct answers to knowledge questions			
	Diagnosis	N (%)	Management	N (%)
Case 1	Rosacea	254 (89.8%)	Metronidazole Gel or Cream	210 (74.2%)
Case 2	Androgenetic Alopecia	191 (67.5%)	Topical Minoxidil	231 (81.6%)
Case 3	Acute Urticaria	206 (72.8%)	Antihistamine (2nd Generation)	149 (52.7%)
Case 4	Herpes Zoster	236 (83.4%)	Acyclovir	231 (81.6%)
Case 5	Atopic Dermatitis	202 (71.4%)	Topical Glucocorticoids	246 (86.9%)
Case 6	Tinea Corporis	133 (47.0%)	Itraconazole	125 (44.2%)
Case 7	Pityriasis Versicolor	174 (61.5%)	Miconazole cream	173 (61.1%)
Case 8	Tinea Capitis	254 (89.8%)	Oral Terbinafine	163 (57.6%)
Case 9	Seborrheic Keratosis	88 (31.1%)	Reassurance	33 (11.7%)
Case 10	Genital Warts	189 (66.8%)	Cryotherapy	190 (67.1%)

Regarding the participants' ability to deal with dermatological cases, about 72% totally agreed or agreed that they usually consult dermatologists regarding some of the ambiguous skin lesions. More than 90% totally agreed or agreed about referring difficult cases to dermatologists. This finding may be attributed to both a lack of confidence in dermatological diagnosis and adherence to institutional referral protocols. In addition, more than 80% acknowledged the importance of continuous training on common skin conditions for PCPs, and that the diagnosis of skin lesions is among the standards of care in primary healthcare (Table 3).

**Table 3:** Participants' answers to questions regarding their ability to deal with dermatological cases.

	Answers to the ability to deal with dermatological cases questions n (%)				
Statement	Totally agree	Agree	Neutral	Disagree	Totally disagree
“I feel capable of diagnosing and managing common skin conditions encountered within my primary healthcare center.”	49 (17.3%)	130 (45.9%)	90 (31.8%)	13 (4.59%)	1 (0.35%)
“I usually discuss skin conditions with my colleagues within the center before managing the patient.”	60 (21.2%)	117 (41.3%)	83 (29.3%)	19 (6.71%)	4 (1.41%)
“I usually consult dermatologists regarding some of the ambiguous skin lesions that are not familiar to me.”	85 (30.0%)	121 (42.8%)	51 (18.0%)	23 (8.13%)	3 (1.06%)
“I usually refer difficult skin conditions to ensure the utmost care of the patient under the hands of Dermatologists.”	135 (47.7%)	121 (42.8%)	24 (8.48%)	3 (1.06%)	0 (0%)
“Offering continuous training on common skin conditions is essential for primary healthcare providers.”	168 (59.4%)	90 (31.8%)	22 (7.77%)	3 (1.06%)	0 (0%)
“Enhanced skin lesion-targeted education in family medicine residency training programs help in early detection of skin cancer during screening.”	147 (51.9%)	103 (36.4%)	30 (10.6%)	2 (0.71%)	1 (0.35%)
“Diagnosing and detecting skin lesions are the standards of care in primary healthcare.”	123 (43.5%)	125 (44.2%)	32 (11.3%)	3 (1.06%)	0 (0%)

The total knowledge score ranged between 4 and 20, with a median of 13 (interquartile range of 10-16). The participants were divided into two groups based on the median total score: those with high knowledge (n = 134) and those with low knowledge (n = 149). High knowledge level was significantly associated with older age (p<0.001), being a consultant or specialist (p<0.001), and working in a primary healthcare center (p<0.001). Interestingly, a high knowledge level was significantly associated with a lower weekly rate of dermatological cases (Table 4).

**Table 4:** Association of participants’ characteristics with their knowledge level. ^*1^p<0.05 indicates statistical significance; ^2 ^Chi-squared test for trend in proportions; ^3 ^Pearson’s Chi-squared test; ^4^ Fisher’s exact test.

Characteristics	Overall N = 283	High Knowledge N = 134	Low Knowledge N = 149	P-value
Age, n (%)				<0.001^*1^
Less than 30 years of age	121 (42.8%)	47 (35.1%)	74 (49.7%)	
30-50 years	146 (51.6%)	84 (62.7%)	62 (41.6%)	
More than 50 years of age	16 (5.65%)	3 (2.24%)	13 (8.72%)	
Gender, n (%)				0.3143^*3^
Female	163 (57.6%)	73 (54.5%)	90 (60.4%)	
Male	120 (42.4%)	61 (45.5%)	59 (39.6%)	
Current Position, n (%)				<0.001^*2^
Family Medicine Consultant	45 (15.9%)	32 (23.9%)	13 (8.72%)	
Family Medicine Specialist	70 (24.7%)	48 (35.8%)	22 (14.8%)	
Family Medicine Resident (PGY4/R4)	14 (4.95%)	0 (0%)	14 (9.40%)	
Family Medicine Resident (PGY3/R3)	19 (6.71%)	11 (8.21%)	8 (5.37%)	
Family Medicine Resident (PGY2/R2)	24 (8.48%)	7 (5.22%)	17 (11.4%)	
Family Medicine Resident (PGY1/R1)	23 (8.13%)	5 (3.73%)	18 (12.1%)	
General Practitioner/ Service Resident	88 (31.1%)	31 (23.1%)	57 (38.3%)	
Type of Healthcare Center that you are affiliated with, n (%)				<0.001^*4^
General Hospital	72 (25.4%)	11 (8.21%)	61 (40.9%)	
Primary Healthcare Center	190 (67.1%)	117 (87.3%)	73 (49.0%)	
Private Clinic	1 (0.35%)	0 (0%)	1 (0.67%)	
Private Hospital	20 (7.07%)	6 (4.48%)	14 (9.40%)	
How many dermatological cases are seen within your clinic per week?, n (%)				0.030^*2^
Less than 20 cases per week	191 (67.5%)	100 (74.6%)	91 (61.1%)	
Between 20 and 50 cases per week	83 (29.3%)	31 (23.1%)	52 (34.9%)	
More than 80 cases per week	9 (3.18%)	3 (2.24%)	6 (4.03%)	

Regarding the questions assessing the participants' ability to manage skin cases, the score ranged between 20 and 35, with a median of 29 (interquartile range of 27-31). The participants were divided into two groups based on the median total score: those with high ability (n = 131) and those with low ability (n = 152). There was no significant association between any of the participants' characteristics and their ability to manage dermatological cases (all p-values > 0.05, Table 5).

**Table 5:** Association of participants' characteristics with their ability to deal with dermatological cases. ^1 ^*p<0.05; ^2 ^Chi-squared test for trend in proportions; ^3 ^Pearson’s chi-squared test; ^4 ^Fisher’s exact test.

Characteristics	Overall N = 283	Low ability N = 152	High ability N = 131	P-value
Age, n (%)				0.564^2^
Less than 30 years of age	121 (42.8%)	67 (44.1%)	54 (41.2%)	
30-50 years	146 (51.6%)	76 (50.0%)	70 (53.4%)	
More than 50 years of age	16 (5.65%)	9 (5.92%)	7 (5.34%)	
Gender, n (%)				0.273^3^
Female	163 (57.6%)	83 (54.6%)	80 (61.1%)	
Male	120 (42.4%)	69 (45.4%)	51 (38.9%)	
Current Position, n (%)				0.122^2^
Family Medicine Consultant	45 (15.9%)	22 (14.5%)	23 (17.6%)	
Family Medicine Specialist	70 (24.7%)	32 (21.1%)	38 (29.0%)	
Family Medicine Resident (PGY4/R4)	14 (4.95%)	10 (6.58%)	4 (3.05%)	
Family Medicine Resident (PGY3/R3)	19 (6.71%)	10 (6.58%)	9 (6.87%)	
Family Medicine Resident (PGY2/R2)	24 (8.48%)	17 (11.2%)	7 (5.34%)	
Family Medicine Resident (PGY1/R1)	23 (8.13%)	13 (8.55%)	10 (7.63%)	
General Practitioner/ Service Resident	88 (31.1%)	48 (31.6%)	40 (30.5%)	
Type of healthcare center that you are affiliated with, n (%)				0.329^4^
General Hospital	72 (25.4%)	42 (27.6%)	30 (22.9%)	
Primary Healthcare Center	190 (67.1%)	102 (67.1%)	88 (67.2%)	
Private Clinic	1 (0.35%)	0 (0%)	1 (0.76%)	
Private Hospital	20 (7.07%)	8 (5.26%)	12 (9.16%)	
How many dermatological cases are seen within your clinic per week?, n (%)				0.499^2^
Less than 20 cases per week	191 (67.5%)	105 (69.1%)	86 (65.6%)	
Between 20 and 50 cases per week	83 (29.3%)	42 (27.6%)	41 (31.3%)	
More than 80 cases per week	9 (3.18%)	5 (3.29%)	4 (3.05%)	

**Figure 1:**
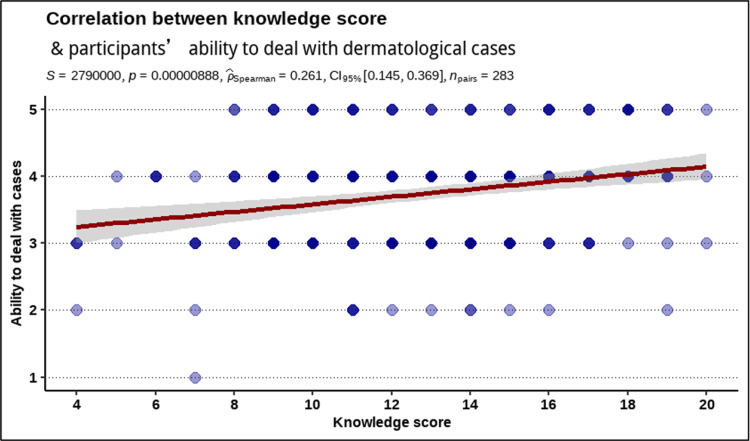
Spearman’s rank-order correlation between total knowledge score and the participant’s ability to deal with dermatological cases.

The correlation between the total knowledge and ability Likert score was weak (rho = 0.261 [95% confidence interval: 0.145, 0.369], p = 0.002, Figure 1).

## Discussion

The present study aims to assess the knowledge of PCPs in diagnosing and treating common dermatological conditions.

We found that most of the respondents' answers to knowledge questions were correct. However, more than half of the respondents did not know the diagnosis and treatment of tinea corporis and seborrheic keratosis. Moreover, 47.3% and 42.4% of the respondents did not know the correct treatment for acute urticaria and tinea capitis, respectively. These results indicate serious gaps in the PCPs' knowledge, as these skin conditions are among the most commonly encountered disorders. A high knowledge level was significantly associated with older age (p<0.001), being a consultant or specialist (p<0.001), and working in a primary healthcare center (p<0.001). The surprising observation that higher levels of knowledge were associated with lower weekly rates of dermatological cases may be explained by multiple factors. One potential explanation is that less confident PCPs may refer more cases to dermatologists, thereby limiting their hands-on experience in managing skin conditions. Additionally, more knowledgeable PCPs, such as consultants and specialists, may work in settings where they encounter fewer dermatology cases due to different clinical responsibilities. These factors suggest the possibility of self-selection bias, which should be explored in future research.

Previous studies have reported varying results regarding the knowledge of PCPs on common skin disorders. Bahelah et al. [10] from Yemen found serious gaps in the knowledge of PCPs, similar to those detected in the present study. They reported that 87.5% of the included PCPs believed that glucocorticoids are the first-line therapy for anaphylactic reactions, and that 50% did not know the right treatment for fungal infections [10].

Al-Zahrani et al. [11] reported that 69.5% of the included PCPs from Abha City, Saudi Arabia, had insufficient knowledge about common skin diseases. Similar to our results, they found a significant association between higher knowledge levels and having higher postgraduate degrees and years of work experience [11]. Moreover, Alotaibi et al. [12] found that 83.6% of their respondents had acceptable to good knowledge. They similarly reported that higher knowledge was associated with postgraduate degrees and years of experience. A study from Western India by Thakkar et al. [9] found that 88.5% of their participants scored less than 50% marks on identifying skin lesions and their treatment.

The insufficient knowledge of PCPs reported by the current research and previous studies could be explained by the inadequate focus on dermatology in the undergraduate curricula of medical students compared to other branches of medicine. The study by Hansra et al. [13] in the United States reported that nearly 40% of PCPs felt that the undergraduate medical curriculum they undertook did not adequately prepare them to manage common skin disorders.

The present study explored the participants' ability to deal with dermatological cases. The results showed that nearly 63.2% were confident that they could diagnose and manage common skin conditions encountered within their primary healthcare center. The correlation between agreement to this statement and overall knowledge was significant and positive, yet weak, reflecting the non-concordance between the PCPs' actual knowledge and their belief about their competence.

In partial agreement with our findings, Bahelah et al. [10] reported the lack of a relationship between the PCPs’ ability to identify skin lesions and their self-perception of competency in dermatology. Moreover, Thakkar et al. [9] detected a poor correlation between the physicians’ score of knowledge and the self-rating of their competence to manage common skin disorders.

The lack of correlation between the actual knowledge level of PCPs and their self-perception of their abilities has been reported by previous studies in different aspects of primary healthcare; thus, this finding was not limited to the field of dermatological disorders. Tracey et al. [14] found a poor correlation between the general practitioners’ self-evaluation and their test scores. In addition, Evans et al. [15] reported that physicians' self-perception may not correlate with their actual clinical competence.

Al-Zahrani et al. [11] reported that about one-third of the respondents were very confident in managing acne (32.4%) and eczema (31.4%), compared to only 13.3%, 11.4%, and 10.5% of PCPs who were very confident in managing fungal diseases, warts, and rosacea, respectively. This suggests that physicians’ perceptions of their ability to manage common skin disorders differ from one dermatological condition to another.

Another important consideration is the referral rate to dermatology clinics, as PCPs are supposed to act as gatekeepers to manage commonly encountered non-serious conditions, thereby reducing the referrals and the burden on specialized clinics. However, 72% of our participants agreed that they usually consult dermatologists regarding some ambiguous skin lesions. In addition, more than 90% agreed about the referral of difficult cases. This seems to contradict the expected role of PCPs in decreasing unjustified referrals and indicates the need to improve the competence of PCPs in managing common skin disorders through training programs.

Other factors may contribute to the increased referral of patients to specialists, besides the level of physicians' knowledge. Alotaibi et al. [12] reported that other factors such as the supporting infrastructure of primary healthcare centers and regulatory issues may play an important role in increasing unjustified referrals to dermatology clinics.

The results of the current study also showed that more than 80% of the respondents acknowledged the importance of continuous training on common skin conditions for PCPs and considered that the diagnosis of skin lesions is among the standards of care in primary healthcare. Likewise, Kerr et al. [16] reported that about 80% of PCPs felt the need for training in dermatology and recommended that training should be followed by regular updates. Thakkar et al. [9] mentioned that PCPs believed that attending dermatology training can help them reduce the difficulties they face in managing dermatological disorders.

The impact of training was depicted in the study by Al-Hoqail et al. [17] in Saudi Arabia, who found that PCPs who received short clinical training in dermatology were better at diagnosing and treating skin disorders compared to their counterparts who did not attend training. In addition, Al-Zahrani et al. [11] found a significantly higher percentage of physicians who had training courses in dermatology had sufficient knowledge compared to those who did not have training (51.7% vs. 22.4%, respectively, p=0.003). Appropriate training in dermatology can enable PCPs to reach more precise diagnoses and improve their competence in managing skin conditions [1,16,18,19].

Dermatologists can also play a pivotal role in elevating the level of knowledge and practice of PCPs who deal with skin disorders. Harlow and Burton [20] surveyed a sample of general practitioners in the United Kingdom and suggested that the dermatology department could help PCPs through the launching of continuing education programs and the creation of clinical practice guidelines that primarily target PCPs.

To enhance the dermatological competence of PCPs in Saudi Arabia, it is essential that medical curricula incorporate more comprehensive dermatology training. Additionally, continuing medical education programs should offer regular updates on dermatological conditions. Furthermore, integrating telemedicine consultations with dermatologists can facilitate real-time case discussions and education. Practical dermatology training must be an integral component of medical education to equip PCPs with the necessary skills to diagnose and manage common skin conditions effectively. Hands-on workshops and structured CME programs can further enhance their expertise and help minimize unnecessary referrals to dermatologists.

The current study showed several points of strength as it included PCPs from various provinces in Saudi Arabia with varying grades of experience, and the cases in the questionnaire encompassed common skin disorders that are usually present in primary healthcare practice. The study also identified gaps in the knowledge of PCPs that should be addressed and explored the factors that affect the PCPs' knowledge level. Meanwhile, the present study showed some limitations. Some serious skin disorders, such as skin cancers, were not included in the photo quiz. In addition, the questionnaire did not ask about attendance at specific training in dermatology, so we were unable to assess the impact of training on knowledge level. Furthermore, the study relied on self-reported confidence levels, which may be influenced by social desirability bias or overestimation of ability. Future studies should consider objective clinical assessments, such as direct observation or standardized patient interactions, to provide a more accurate evaluation of PCPs’ dermatology skills.

## Conclusions

The knowledge level of PCPs regarding the management of common skin disorders shows serious deficiencies. The PCPs’ beliefs demonstrate an inclination toward unnecessary referrals to dermatologists. The implementation of specific training programs in dermatology for PCPs is warranted. Additionally, dermatology courses in medical school curricula should prepare students to effectively manage common dermatological conditions.
